# Attention-Deficit/Hyperactivity Disorder Traits in Childhood and Physical Health in Midlife

**DOI:** 10.1001/jamanetworkopen.2025.54802

**Published:** 2026-01-21

**Authors:** Joshua Stott, Elizabeth O’Nions, Lucy Corrigan, Joanne Cotton, Warren James Donnellan, Danielle Nimmons, Henry Shelford, Céline El Baou, Gavin R. Stewart, Rachael W. Cheung, Roopal Desai, Douglas G. J. McKechnie, Aphrodite Eshetu, Rob Saunders, Jae Won Suh, William Mandy, Darya Gaysina, Philip Asherson, Jessica Agnew-Blais, Amber John

**Affiliations:** 1 Clinical, Educational, and Health Psychology, University College London, London, United Kingdom; 2 Bradford Institute for Health Research, Bradford Teaching Hospitals NHS Foundation Trust, Bradford, United Kingdom; 3 Department of Psychology, University of Liverpool, Liverpool, United Kingdom; 4 Independent researcher; 5 Department of Primary Care and Population Health, University College London, United Kingdom; 6 ADHD UK, London, United Kingdom; 7 Social, Genetic and Developmental Psychiatry Centre, Kings College London, London, United Kingdom; 8 School of Psychology, University of Sussex, Brighton, United Kingdom; 9 Department of Psychology, Queen Mary University of London, London, United Kingdom

## Abstract

**Importance:**

Although attention-deficit/hyperactivity disorder (ADHD) has been associated with various health risks, most research has focused on children and young adults, leaving long-term physical health outcomes of ADHD traits underexplored.

**Objective:**

To investigate the association between childhood ADHD traits and physical health outcomes in midlife and the role health risk factors play in this association.

**Design, Setting, and Participants:**

This cohort study included data from the nationally representative, population-based 1970 British Cohort Study. The cohort included people born in England, Scotland, and Wales during the same week in 1970, with follow-up data collected over 46 years. Participants were excluded from the analysis if missing data on 1 or more key variables (sex, ADHD traits, social class, and ethnicity) assessed. The data analysis was performed between February and July 2025.

**Exposure:**

Attention-deficit/hyperactivity disorder traits at age 10 years as assessed using a validated measure derived from childhood behavior questionnaires.

**Main Outcomes and Measures:**

Outcomes were self-reported health conditions, multimorbidity (2 or more co-occurring physical health conditions), and physical health–related disability by age 46 years. Cox proportional hazards models were used to test whether ADHD traits at age 10 years were associated with hazards of multimorbidity up to age 46 years. Health risk factors, including smoking, alcohol use, psychological distress, higher body mass index, and lower educational attainment from age 26 to 46 years, were examined using path models.

**Results:**

A total of 10 930 participants were included in the main analyses (all aged 46 years; 51.0% women). Higher childhood ADHD traits were associated with more physical health conditions (*b* = 0.10; 95% CI, 0.07-0.13), increased odds of physical multimorbidity (odds ratio, 1.14, 95% CI, 1.08-1.19), and greater physical health–related disability (*b* = 3.17; 95% CI, 2.27-4.07) by age 46 years. There were no sex interactions for physical health conditions and multimorbidity. However, the association between ADHD traits and physical health–related disability showed a larger effect size in women (*b* = 4.07; 95% CI, 2.67-5.48) than in men (*b* = 2.37; 95% CI, 1.24-3.51). Participants who had a high likelihood of meeting ADHD criteria in childhood (5.5%) had an estimated probability of 42.1% (95% CI, 38.2%-46.1%) of physical multimorbidity by age 46 years compared with 37.5% (95% CI, 36.6%-38.4%) for those without high ADHD traits. Indirect associations were observed through smoking, psychological distress, and body mass index.

**Conclusions and Relevance:**

This cohort study found that high ADHD traits in childhood were associated with poorer physical health outcomes in midlife, with health risk factors explaining part of this association. Addressing modifiable risk factors may help mitigate long-term health disparities in people with ADHD. Intervention is needed across the life course to support the health and well-being of people with ADHD.

## Key Points

**Question:**

Are childhood attention deficit/hyperactivity disorder (ADHD) traits associated with physical health outcomes in midlife, and do health risk factors modify the associations?

**Findings:**

In this cohort study of 10 930 participants, more childhood ADHD traits were associated with a greater number of physical health problems, increased risk of physical multimorbidity, and more physical health-related disability by age 46 years. These associations were partially explained by smoking, psychological distress, and body mass index.

**Meaning:**

These findings suggest that early ADHD traits are associated with poorer long-term physical health, highlighting the importance of early identification and targeted support across the life course.

## Teaser

This cohort study investigates the association between childhood attention-deficit/hyperactivity disorder (ADHD) traits and physical health outcomes in midlife and the role health risk factors play in this association.

## Introduction

Attention-deficit/hyperactivity disorder (ADHD) is a neurodevelopmental condition characterized by persistent inattention, hyperactivity, and/or impulsivity, which occurs in multiple contexts and interferes with daily functioning.^1^ It has historically been viewed as a childhood condition, but evidence has shown that ADHD continues into adulthood for many children with the condition.^2,3^ It should be noted that people with ADHD are a highly heterogeneous group and that experiences vary substantially depending on a wide range of factors (eg, age, sex, geography, access to support).

There is growing recognition that many people with ADHD disproportionately face cumulative disadvantage and adversity over the course of their lives, shaped by a range of individual, social, and structural factors,^4,5,6,7^ which may contribute to reduced life expectancy.^8^ People with ADHD are at increased risk of adverse health outcomes,^9^ including metabolic disorders,^10^ cardiovascular diseases,^11^ nonsuicidal self-injury,^12^ suicidal behavior,^13,14^ and premature mortality.^8^ Health risk factors, such as smoking and harmful alcohol use,^15,16,17,18^ are more common in people with ADHD, as well as mental health symptoms, psychological distress,^19,20,21^ and obesity.^22^ Educational disadvantages are also disproportionately experienced by people with ADHD,^23,24^ limiting access to stable employment, financial security, and health care.

Research into health outcomes for middle-aged (40-65 years) and older (≥65 years) adults with ADHD has often focused on people with an ADHD diagnosis. Well-documented barriers to clinical recognition mean that many people with ADHD are undiagnosed, and diagnosed groups may overrepresent individuals with more co-occurring health conditions and support needs.^8,25^ As such, relying on diagnosis alone may bias results. Longitudinal population-based studies using ADHD measures collected in childhood could model the association of childhood ADHD traits with later health outcomes.

Most prior research on ADHD and physical health has focused on associations with individual conditions. The presence of multiple co-occurring health conditions is associated with increased disability,^26,27^ reduced quality of life,^28,29^ and higher health care use and costs.^30^ Reducing these health conditions is a key priority for public health and policy, which may be particularly relevant to ADHD given the broad range of physical and mental health problems associated with the condition and evidence that people with ADHD experience socioeconomic adversity and barriers to health care access.^9,31^ The aim of our study was to assess associations between ADHD traits measured in childhood and physical health outcomes in midlife using data from a longitudinal cohort with follow-up data spanning birth to middle age.

## Methods

### Data Source

This cohort study used data from the 1970 British Cohort Study (BCS70),^32^ which includes 17 198 people born in England, Scotland, and Wales during 1 week of 1970, with follow-up data collected over 46 years. The BCS70 collects a wide range of demographic data and information on health and lifestyle factors. Detailed information about data collection and participation rates are published elsewhere.^33,34^ Ethical approval for the most recent wave of data collection was obtained from the South East Coast–Brighton and Sussex Medical Research Ethics Committee. Parents (for childhood sweeps) and cohort members (for adulthood sweeps) provided written informed consent. The study followed the Strengthening the Reporting of Observational Studies in Epidemiology (STROBE) reporting guideline.

### Measures

#### ADHD Traits

A measure of ADHD traits was derived based on parent and teacher responses to items from child behavior questionnaires administered in 1980 when the BCS70 cohort was aged 10 years.^35,36^ At this time, clinical diagnoses of ADHD were very uncommon in the UK due to limited clinical and public recognition and different diagnostic practices. The 14-item measure (9 items corresponding to hyperactivity and 5 to inattention) of ADHD traits was derived in previous research, using a data mining framework to map items to ADHD symptoms in the *Diagnostic and Statistical Manual of Mental Disorders *(Fifth Edition) (*DSM-5*). A dimensional score of ADHD traits was derived using a zero-inflated item response theory mixture model based on these items, as well as a binary score indicating whether *DSM-5* criteria for ADHD were met.^37^ Detailed information about these measures is published elsewhere,^37^ with a user guide available from the Centre for Longitudinal Studies. Items are presented in eTable 1 in Supplement 1.

This approach has good model fit and discriminative validity, with expected proportions in the high ADHD traits group (5.5%). The scale is associated with established correlates of ADHD, including male sex, social disadvantage, and educational and behavioral outcomes.^37^ The dimensional score was used in the main analyses.

#### Physical Health Problems and Disability

Measures of physical health problems were self-reported over the course of adult life in BCS70 between age 26 and 46 years. Previous research has found that self-reported health measures can reliably estimate the prevalence of chronic diseases.^38,39^ Physical health conditions measured at the majority of available time points were selected for inclusion and were asthma or wheezy bronchitis; migraine; backache or back problems; cancer or leukemia; epilepsy or seizure; diabetes; hearing problems; problems with stomach, bowels, or gallbladder; and problems with bladder or kidneys. For each condition, a binary score was derived that indicated whether the condition was reported. Use of these measures is consistent with approaches used in previous research.^40^

The primary outcome was physical multimorbidity. Physical multimorbidity was captured using a binary measure that indicated whether participants reported 2 or more health conditions by age 46 years and at what age they were first reported.

Secondary outcomes were the number of health problems and physical health–related disability. A continuous measure was created that indicated the number of distinct physical health problems participants reported by age 46 years (range, 0-9). Physical health–related disability at age 46 years was measured using the role limitations due to physical health subscale from the 36-Item Short Form Survey.^41^ Participants were asked whether they had problems with work or other regular daily activities as a result of their physical health during the past 4 weeks, including 4 items related to limitations due to physical health. Response options for each item were on a binary scale of yes or no. A summary score was derived out of 100, indicating level of disability as a result of physical health.

#### Health Risk Factors

Health risk factors considered as indirect pathways in this study were smoking, psychological distress, alcohol use, high body mass index (BMI), and low educational attainment. Longitudinal measures of cumulative exposure to health risk factors across early adulthood to midlife (age 26, 30, 34, 38, 42, and 46 years) were derived for each measure. The proportion of valid time points in which participants met criteria for the health risk was calculated instead of summed scores to maximize sample size for individuals with missing data. People with data available at fewer than 3 time points were excluded from this calculation. Detailed information about questionnaires and items used to derive cumulative scores for each health risk factor is provided in the eMethods in Supplement 1.

### Covariates

Covariates were sex, ethnicity, and social class at age 10 years. Sex was coded as female or male, and parent-reported ethnicity as European White or ethnic minority (combined due to low numbers and included Bangladeshi; English, Northern Irish, Scottish, or Welsh; Indian; Irish; other European; Pakistani; West Indian or Guyanese; mixed parentage; or any other ethnic group) to account for potential confounding associated with ethnicity. The measure of social class at age 10 years was based on the father’s occupation (or the mother’s occupation if the father’s data were missing) and coded according to the Registrar General’s Social Class schema (unskilled, partly skilled, manual, nonmanual, managerial and technical, and professional).^42,43^

### Statistical Analysis

The BCS70 participants with missing data were compared with those with complete data to test whether they differed on the key variables based on *t* tests and χ^2^ tests. Logistic regressions were performed to test associations between ADHD traits at age 10 years and multimorbidity by age 46 years. Probabilities of multimorbidity by age 46 years were estimated using postestimation marginal effects (the marginal command in Stata, version 18 [StataCorp LLC]) following logistic regression, comparing people with and without high ADHD traits using the binary indicator.^37^ Linear regressions were performed to test associations between ADHD traits and the number of physical health problems reported and physical health–related disability at age 46 years. Interactions with sex were modeled to test whether stratified models were needed.

Cox proportional hazards models were used to test whether ADHD traits at age 10 years were associated with hazards of multimorbidity up to age 46 years. Time to event was measured in years from the start of follow-up (age 26 years). Participants who reported multimorbidity at the start of follow-up were excluded from analyses. Participants were censored at death, permanent emigration, permanent study dropout, or the latest data collection point (age 46 years), whichever came first. The assumption of proportional hazards was checked using Schoenfeld residuals.

Path models were used to test indirect associations of ADHD traits with (1) multimorbidity and (2) physical health–related disability at age 46 years through health risk factors. Covariances were included between health risks to account for known correlations.

Analyses were performed unadjusted and then adjusted for covariates. Analyses for this study were performed between February and July 2025, using MPlus, version 8 (Muthén and Muthén) and Stata, version 18. Missing data were evaluated using full information maximum likelihood.^44^ A 2-sided *P* < .05 was considered statistically significant.

## Results

### Descriptive Statistics and Missing Data

A total of 10 930 participants were included in the main analyses (all aged 46 years at latest follow-up; 49.0% men and 51.0% women; 96.8% of White ethnicity and 3.2% of an ethnic minority group) (Table 1). These participants were compared with those excluded from the model due to missing data (n = 5113) on 1 or more key variables or covariates required for analysis. Key differences between samples were observed and are shown in eTable 2 in Supplement 1. The breakdown of health problems reported by condition is presented in eTable 3 in Supplement 1.

**Table 1.** Characteristics of the Sample Included in the Main Models (N = 10 930)

Characteristic	Participants, No. (%)
ADHD traits, mean (SD)^a^	−0.09 (0.86)
Multimorbidity	
No	6807 (62.3)
Yes	4123 (37.7)
No. of physical health problems, mean (SD)	1.3 (1.2)
Physical health–related disability score, mean (SD)^b^	14.9 (30.9)
Proportion current smoking, mean (SD)^c^	0.3 (0.4)
Proportion high alcohol use, mean (SD)^c^	0.2 (0.3)
Proportion high BMI, mean (SD)^c^	0.5 (0.4)
Proportion high psychological distress, mean (SD)^c^	0.2 (0.3)
Education	
No qualifications	1954 (27.4)
GCSEs or equivalent	2300 (32.2)
Advanced Subsidiary or Advanced levels or equivalent	404 (5.7)
Diploma, degree, or higher degree	2480 (34.7)
Sex	
Female	5573 (51.0)
Male	5357 (49.0)
Ethnicity	
White	10 584 (96.8)
Ethnic minority group^d^	346 (3.2)
Social class at age 10 years^e^	
Unskilled	433 (4.0)
Partly skilled	1460 (13.4)
Manual	4476 (41.0)
Nonmanual	1211 (11.1)
Managerial and technical	2672 (24.4)
Professional	678 (6.2)

Abbreviations: ADHD, attention-deficit/hyperactivity disorder; BMI, body mass index; GCSE, General Certificate of Secondary Education.

^a^ Dimensional measure of ADHD traits.

^b^ Scale of 0 to 100, with higher values indicating higher levels of disability as a result of physical health.

^c^ Cumulative measure across adulthood (age 26-46 years).

^d^ Included Bangladeshi; English, Northern Irish, Scottish, or Welsh; Indian; Irish; other European; Pakistani; West Indian or Guyanese; mixed parentage; or any other ethnic group.

^e^ Based on the father’s occupation (or mother’s occupation if the father’s data were missing).

### Childhood ADHD Traits and Physical Health Problems and Disability by Age 46 Years

Attention-deficit/hyperactivity disorder traits were associated with a greater odds of physical multimorbidity by age 46 years (odds ratio [OR], 1.14; 95% CI, 1.08-1.19; *P* < .001) (Table 2). Margins analysis from a logistic regression model using the binary indicator of ADHD traits showed that individuals with high ADHD traits (representing 5.5% of the sample) had an estimated probability of 42.1% (95% CI, 38.2%-46.1%) of experiencing multimorbidity by age 46 years compared with 37.5% (95% CI, 36.6%-38.4%) for those without high ADHD traits. The absolute risk difference was 4.6 percentage points. Attention-deficit/hyperactivity disorder traits were associated with a greater number of health problems (*b* = 0.10; 95% CI, 0.07-0.13; *P* < .001) and greater physical health–related disability reported at age 46 years (*b* = 3.17; 95% CI, 2.27-4.07; *P* < .001).

**Table 2.** Regression Models Testing Associations Between Childhood ADHD Traits and Physical Health Problems and Disability by Age 46 Years

Variable	No. of physical health problems				Multimorbidity				Physical health–related disability			
	Model 1: unadjusted		Model 2: adjusted		Model 1: unadjusted		Model 2: adjusted		Model 1: unadjusted		Model 2: adjusted	
	*b* (95% CI)	*P* value	*b* (95% CI)	*P* value	OR (95% CI)	*P* value	OR (95% CI)	*P* value	*b* (95% CI)	*P* value	*b* (95% CI)	*P *value
ADHD traits	0.05 (0.03 to 0.08)	<.001	0.10 (0.07 to 0.13)	<.001	1.06 (1.02 to 1.10)	.008	1.14 (1.08 to 1.19)	<.001	3.10 (2.25 to 3.95)	<.001	3.17 (2.27 to 4.07)	<.001
Sex												
Female	NA	NA	0.44 (0.39 to 0.48)	<.001	NA	NA	1.93 (1.78 to 2.09)	<.001	NA	NA	5.47 (3.98 to 6.96)	<.001
Male	NA	NA	1 [Reference]	NA	NA	NA	1 [Reference]	NA	NA	NA	1 [Reference]	NA
Ethnicity												
White	NA	NA	1 [Reference]	.23	NA	NA	1 [Reference]	.27	NA	NA	1 [Reference]	NA
Ethnic minority group	NA	NA	−0.08 (−0.21 to 0.05)	NA	NA	NA	0.88 (0.70 to 1.10)	NA	NA	NA	3.39 (−1.02 to 7.80)	.13
Social class at age 10 years	NA	NA	0.001 (−0.02 to 0.02)	.95	NA	NA	1.00 (0.97 to 1.03)	.88	NA	NA	−1.66 (−2.24 to 1.07)	<.001

Abbreviations: ADHD, attention-deficit/hyperactivity disorder; NA, not applicable; OR, odds ratio.

Cox proportional hazards models showed that ADHD traits were associated with increased rates of multimorbidity by age 46 years (hazard ratio, 1.12; 95% CI, 1.07-1.17; *P* < .001) (Table 3). There were no significant interactions between childhood ADHD traits and sex with multimorbidity (OR, 1.03; 95% CI, 0.94-1.12; *P* = .56) or the number of physical health problems (*b* = 0.05; 95% CI, −0.003 to 0.10; *P* = .07), and as such, these models were not run stratified by sex. There was an interaction between childhood ADHD traits and sex on physical health–related disability (*b* = 1.73; 95% CI, 0.01-3.45; *P* = .049). Stratified models showed that ADHD traits were associated with more physical health–related disability in men and women, but with larger effect sizes in women (*b* = 4.07; 95% CI, 2.67-5.48; *P* < .001) compared with men (*b* = 2.37; 95% CI, 1.24-3.51; *P* < .001).

**Table 3.** Cox Proportional Hazards Models Testing Associations Between Childhood ADHD Traits and Multimorbidity by Age 46 Years

Variable	Model 1: unadjusted HR (95% CI)^a^	*P* value	Model 2: adjusted HR (95% CI)	*P* value
ADHD traits	1.09 (1.04-1.13)	<.001	1.12 (1.07-1.17)	<.001
Sex				
Female	NA	NA	1.45 (1.34-1.58)	<.001
Male	NA	NA	1 [Reference]	
Ethnicity				
White	NA	NA	1 [Reference]	.51
Ethnic minority group	NA	NA	0.93 (0.74-1.16)	
Social class at age 10 years	NA	NA	0.97 (0.94-1.00)	.08

Abbreviations: ADHD, attention-deficit/hyperactivity disorder; HR, hazard ratio; NA, not applicable.

^a^ Proportional hazards assumption not violated: χ^2^_1_ = 1.42; *P* = .23.

### Health Risk Factors Underlying Associations of Childhood ADHD Traits With Physical Health Problems and Disability

**Figure 1.**
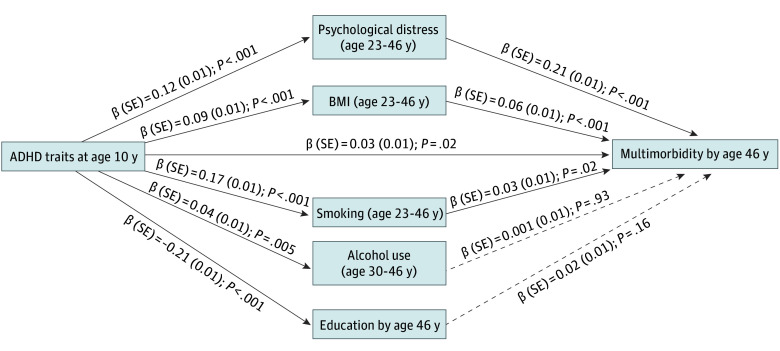
Path Model Testing the Role of Health Risk Factors in the Association Between Childhood Attention-Deficit/Hyperactivity Disorder (ADHD) Traits and Multimorbidity by Age 46 Years The model included 10 930 individuals and was adjusted for sex, ethnicity, and social class at age 10 years. Solid lines indicate a significant association at *P* < .05, and dashed lines indicate no association.

The path model that tested the role of health risk factors in the association between ADHD traits at age 10 years and multimorbidity among all participants by age 46 years showed a direct association of ADHD traits with multimorbidity (β [SE], 0.03 [0.01]; *P* = .02) (Figure 1). There were also indirect associations of ADHD with multimorbidity through smoking (β [SE], 0.01 [0.002]; *P* = .02), BMI (β [SE], 0.01 [0.001]; *P* < .001), and psychological distress (β [SE], 0.03 [0.003]; *P* < .001). Although ADHD traits were associated with higher alcohol use (β [SE], 0.04 [0.01]; *P* = .005) and lower educational attainment (β [SE], −0.21 [0.01]; *P* < .001), these health risk factors were not associated with multimorbidity.

**Figure 2.**
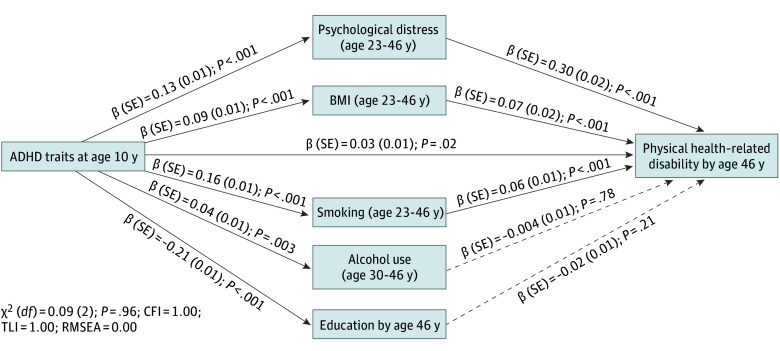
Path Model Testing the Role of Health Risk Factors in the Association Between Childhood Attention-Deficit/Hyperactivity Disorder (ADHD) Traits and Physical Health–Related Disability at Age 46 Years The model included 9268 individuals and was adjusted for sex, ethnicity, and social class at age 10 years. Solid lines indicate a significant association at *P* < .05, and dashed lines indicate no association. CFI indicates comparative fit index; RMSEA, root mean square error of approximation; TLI, Tucker-Lewis index.

The path model that tested the role of health risk factors in the association between ADHD traits at age 10 years and physical health–related disability at age 46 years (n = 9268) showed a direct association of ADHD traits with disability (β [SE], 0.03 [0.01]; *P* = .02) (Figure 2). There were also indirect association of ADHD with disability through smoking (β [SE], 0.01 [0.002]; *P* < .001), BMI (β [SE], 0.01 [0.001]; *P* < .001), and psychological distress (β [SE], 0.04 [0.004]; *P* < .001). Attention-deficit/hyperactivity disorder traits were associated with higher alcohol use (β [SE], 0.04 [0.01]; *P* = .003) and lower educational attainment (β [SE], −0.21 [0.01]; *P* < .001), but these were not associated with physical health-related disability.

## Discussion

This cohort study used the large, population-based BCS70 cohort with follow-up over several decades to examine the association between childhood ADHD traits and physical health problems in midlife. The study found that ADHD traits in childhood are associated with poorer physical health outcomes in midlife, including more physical health conditions, increased risk of multimorbidity, and greater physical health–related disability by age 46 years. Participants with high ADHD traits in childhood had an estimated probability of multimorbidity of 42.1% by age 46 years compared with 37.5% for those without high ADHD traits.

Our results align with previous research suggesting that ADHD is associated with health risks, including smoking, alcohol use, high BMI, psychological distress, and lower educational attainment.^15,16,17,18,21,22,23,24^ They extend previous research by indicating that cumulative exposure to modifiable health risk factors partly underpin associations between ADHD and multimorbidity and physical disability. However, direct associations between ADHD and physical health outcomes remained significant, suggesting that other mechanisms are also important (eg, social exclusion, accidental injury, barriers to accessing timely appropriate medical care). Overall, the findings highlight the need for a life course perspective on ADHD and its associated health inequities and suggest that avoidable morbidity in people with ADHD should be addressed.

Future research should explore strategies for early health screening and prevention to improve long-term health outcomes for people with ADHD. Given the role of social exclusion (eg, unemployment, social isolation) in shaping health outcomes, future work should consider how addressing these broader determinants could improve overall health and well-being for adults with ADHD. Participants excluded from these analyses due to missing data were more likely to be from ethnic minority backgrounds, have a lower social class, and have higher ADHD traits at age 10 years. Given that both ethnicity and deprivation are associated with poor health,^45^ people with high ADHD traits from these groups may be at high risk of additional morbidity. Future research should focus efforts on reducing attrition in underserved communities to provide a better evidence base for health screening and prevention. Studies should investigate potential adaptations to interventions targeting lifestyle factors to make them more inclusive and accessible for people with ADHD and mitigate the increased risk of chronic conditions. Interventions should be codesigned in collaboration with people with ADHD and should consider universal design principles to reduce stigma and promote wider engagement.

The results have important implications for public health and clinical practice. They suggest that early detection and ongoing support is needed for people with ADHD across the lifespan. Clinicians should be aware of the increased rates of physical health problems and associated disability in people with ADHD and should proactively address potential contributing health risk factors. People with ADHD may face more barriers to attending routine screening programs (eg, cervical cancer screening) and monitoring for chronic conditions (eg, diabetes checks), which may contribute to worsening of physical health and greater disability. Services should be aware of these barriers and ensure that routine screening and monitoring programs are accessible. Public health interventions targeting key health risks (eg, focusing on smoking cessation, weight management, and improvement of mental health symptoms) may help reduce some of these negative health consequences. Further research is needed to understand the impacts of such interventions. Integrated interventions addressing mental health, physical health, and key health risk factors may help to reduce chronic conditions in this population. Clinicians should be aware of potential long-term health risks associated with ADHD, which may improve early screening for health problems and preventive care strategies. Improved training on the association between ADHD and physical health for clinicians in primary and secondary care, alongside increased resources for integrated community care such as increased appointment times,^46^ may help make improved care for people with ADHD achievable.

### Strengths and Limitations

Attention-deficit/hyperactivity disorder traits were assessed using a dimensional trait-based measure based on the *DSM-5*, which has shown good psychometric properties. Thus, our study was not reliant on diagnosis data given that ADHD is underdiagnosed,^8,25^ and at the time of this assessment, diagnoses would have been very uncommon. The prospective nature of data collection means that recall bias did not influence the findings.

This study also had several limitations. First, reliance on self-reported health conditions may have introduced reporting or recall biases, leading to potential misclassification of physical health problems. Although previous research has shown that self-reported health measures can provide valid estimates of chronic disease prevalence,^38,39^ these factors should be considered when interpreting findings.

Second, the measure of multimorbidity included only 9 physical health conditions, meaning that other important conditions were not considered in these analyses, including cardiovascular disease, which may be significantly associated with ADHD. The lack of inclusion of such conditions may have contributed to the relatively modest effect sizes observed in this study. The use of physical health–related disability as a secondary outcome helped to address this limitation, as it captured role limitations and disability due to physical health issues, regardless of their specific causes. Results were consistent between these 2 outcomes.

Third, although several key health risk factors and covariates were accounted for in models, other possible factors were not considered (eg, health care access or experiences, social or societal factors, medications). Medication for ADHD may plausibly affect physical health risks through both direct pharmacologic effects and indirect pathways (eg, improved symptom management and health care engagement), but the overall balance of risks and benefits remains unclear. Although psychological distress was included in models as a potential pathway, the study did not have symptom measures for specific mental health problems (eg, depression, anxiety). As such, it was not possible to examine the influences of particular co-occurring mental health problems separately. Future research should explore possible mediators and moderators in more detail, given the heterogeneity within ADHD populations.

Fourth, people from ethnic minority groups were underrepresented in this sample. Although the cohort was broadly representative of the UK population during 1970, it no longer reflects the diversity of the contemporary population, meaning that results are not applicable to more diverse populations.

Finally, there was substantial attrition in this sample, and people with high ADHD traits, greater physical disability, and higher social disadvantage were more likely to drop out of the study. Thus, people most at risk of poor health outcomes may be underrepresented, which may lead to an underestimation in the findings. Missing data were accounted for using full information maximum likelihood; however, this does not completely eliminate the issue as the participants excluded from the analytic sample differed from those included on key characteristics, including ADHD traits, social class, and ethnicity.

## Conclusions

This cohort study found that high ADHD traits in childhood is associated with poorer physical health outcomes in midlife, partly through cumulative exposure to health risk factors across adulthood. Women may experience more physical health–related disability associated with childhood ADHD traits. Although effect sizes were relatively modest, these findings highlight key opportunities for providing better support to people with ADHD. It is important to acknowledge that people with ADHD are a diverse group with a range of different experiences. Many people with ADHD lead long, healthy lives. Improving understanding of protective factors and estimators of resilience in these groups may help to inform better support systems.

The findings highlight the importance of life course approaches to understanding long-term health problems in people with ADHD. A multidisciplinary approach is needed to tackle these inequities, combining different perspectives (including medical, psychological, and social support). Future research should aim to improve our understanding of mechanisms that underpin observed associations, which may help inform policies and interventions aimed at improving outcomes for people with ADHD over the lifespan.

## Notes

Supplement 1. **eTable 1.** Items Included in Measures of ADHD Traits **eMethods.** Information About Deriving Cumulative Scores of Health Risk Factors
eReferences.
**eTable 2. **Missing Data Analysis **eTable 3. **Health Problems Reported by Age 46 Years by Condition

Supplement 2.
Data Sharing Statement
