# Supplementary material for: Attention-Deficit/Hyperactivity Disorder Traits in Childhood and Physical Health in Midlife
Source: JAMA Netw Open. 2026 Jan 21;9(1):e2554802. doi: 10.1001/jamanetworkopen.2025.54802 (PMC12824776; doi:10.1001/jamanetworkopen.2025.54802)
Supplement: Supplement 1. — eTable 1. Items Included in Measures of ADHD Traits eMethods. Information About Deriving Cumulative Scores of Health Risk Factors eReferences. eTable 2. Missing Data Analysis eTable 3. Health Problems Reported by Age 46 Years by Condition [file jamanetwopen-e2554802-s001.pdf]

Supplemental Online Content

Stott J, O'Nions E, Corrigan L, et al. Attention deficit/hyperactivity disorder traits in childhood and physical health in midlife. *JAMA Netw Open*. 2026;9(1):e2554802.
doi:10.1001/jamanetworkopen.2025.54802

eTable 1. Items Included in Measures of ADHD Traits

eMethods. Information About Deriving Cumulative Scores of Health Risk Factors

eReferences

eTable 2. Missing Data Analysis

eTable 3. Health Problems Reported by Age 46 Years by Condition

This supplemental material has been provided by the authors to give readers additional information about their work.

© 2026 Stott J et al. *JAMA Network Open*.

eTable 1. Items Included in Measures of ADHD Traits

DSM-5 criteria	Corresponding items in BCS70
**Inattentive**	
Often fails to give close attention to details or makes careless mistakes in schoolwork, at work, or with other activities.	N/A
Often has trouble holding attention on tasks or play activities.	R-j155 - Pays attention to what is being explained in class<br>m82 - Has difficulty concentrating on any particular task though may return to it frequently<br>j129 - Cannot concentrate on any particular task, even though the child may return to it frequently<br>j077 - How well does this child concentrate on educational tasks, in comparison with the average 10-year-old?
Often does not seem to listen when spoken to directly.	N/A
Often does not follow through on instructions and fails to finish schoolwork, chores, or duties in the workplace (e.g., loses focus, side-tracked).	m76 - Fails to finish things he/she starts, short attention span<br>R-j174 - Child completes tasks which are started<br>j177 - Fails to finish things he starts
Often has trouble organizing tasks and activities.	N/A
Often avoids, dislikes, or is reluctant to do tasks that require mental effort over a long period of time (such as schoolwork or homework).	R-j139 - Shows perseverance; persists with difficult or routine work
Often loses things necessary for tasks and activities (e.g. school materials, pencils, books, tools, wallets, keys, paperwork, eyeglasses, mobile telephones).	N/A
Is often easily distracted	m65 – Inattentive, easily distracted<br>j152 – Is easily distracted
Is often forgetful in daily activities.	j158 – Is forgetful when given a complex task
**Hyperactive**	

© 2026 Stott J et al. *JAMA Network Open*.

Often fidgets with or taps hands or feet, or squirms in seat.	m44 - Is squirmy or fidgety<br>j151 - Squirmy and fidgety<br>m77 - Given to rhythmic tapping or kicking<br>j165 - Given to rhythmic tapping or rhythmic kicking during class<br>j082 - What percentage of the time is the child fidgeting and indulging other minor distracting activities, when he/she is expected to be working?
Often leaves seat in situations when remaining seated is expected.	j081 - What percentage of the time is the child moving around the classroom, when he/she is expected to be working? (paraphrased)
Often runs about or climbs in situations where it is not appropriate (adolescents or adults may be limited to feeling restless).	m43 - Very restless. Often running or jumping up and down. Hardly ever still.
Often unable to play or take part in leisure activities quietly.	m57 - Cannot settle to do anything for more than a few moments
Is often "on the go" acting as if "driven by a motor".	m72 – Shows restless or overactive behaviour<br>j150 - Shows restless or overactive behaviour
Often talks excessively.	j080 - What percentage of the time is the child talking to other children, when he/she is expected to be working?
Often blurts out an answer before a question has been completed.	m73 – Is impulsive, excitable
Often has trouble waiting his/her turn.	m71 - Requests must be met immediately, easily frustrated<br>j175 - Requests must be met immediately - easily frustrated
Often interrupts or intrudes on others (e.g., butts into conversations or games).	m74 - Interferes with the activity of other children<br>j142 - Interferes with the activities of other children
**Conditional criteria**	
Several inattentive or hyperactive-impulsive symptoms were present before age 12 years.	All criteria evaluated at age 10
Several symptoms are present in two or more settings, (such as at home, school or work; with friends or relatives; in other activities).	If both mother and teacher indicated 4+ symptoms were observed often.

© 2026 Stott J et al. *JAMA Network Open*.

There is clear evidence that the symptoms interfere with, or reduce the quality of, social, school, or work functioning.	N/A
The symptoms are not better explained by another mental disorder (such as a mood disorder, anxiety disorder, dissociative disorder, or a personality disorder).	Excluded if diagnosed with another psychiatric disorder, identified using ICD codes in the medical questionnaire: 295, 296, 300, 301.
The symptoms do not happen only during the course of schizophrenia or another psychotic disorder.	Excluded if diagnosed with another psychiatric disorder, identified using ICD codes in the medical questionnaire: 295, 296, 300, 301.

© 2026 Stott J et al. *JAMA Network Open*.

eMethods. Information About Deriving Cumulative Scores of Health Risk Factors

Measures of **smoking status** were measured at ages 26, 30, 34, 38, 42, and 46. A measure of cumulative smoking was derived by calculating the proportion of valid timepoints in which cohort members reported being a current smoker.

Measures of **alcohol use** were available at ages 30, 34, 42, and 46, using the CAGE Questions for Alcohol Use[1] (ages 30 and 34) and the Alcohol Use Disorders Identification Test (AUDIT)[2,3] (ages 42 and 46). Cohort members were categorised as having high alcohol use if they scored over the threshold for these questionnaires (≥2 for CAGE; ≥5 AUDIT). A measure of cumulative alcohol use was derived by calculating the proportion of valid timepoints in which cohort members scored over these thresholds.

Psychological distress was measured at ages 26, 30, 34, 42, and 46, using the Malaise Inventory Scale[4,5]. To ensure consistency, the self-report short-form (9 items) was used at all available timepoints. Cohort members were categorised as having high psychological distress levels if they scored over the clinical threshold for case-level symptoms (≥4). A measure of cumulative distress was derived by calculating the proportion of valid timepoints in which cohort members reported high psychological distress levels.

Measures of **BMI** were derived from height and weight measurements at ages 26, 30, 34, 42, and 46. At each timepoint, cohort members were classified as overweight if their BMI was ≥25. A measure of cumulative BMI was derived by calculating the proportion of valid timepoints in which cohort members were classified as ‘overweight’.

Finally, **education** was derived based on the highest academic qualification achieved by age 46 (No qualifications, GCSE/O-Levels, AS/A-Levels, diploma/degree or higher).

eReferences

1. Ewing JA. Detecting alcoholism: the CAGE questionnaire. *JAMA*. 1984;252(14):1905-1907.
2. Saunders JB, Aasland OG, Babor TF, De la Fuente JR, Grant M. Development of the alcohol use disorders identification test (AUDIT): WHO collaborative project on early detection of persons with harmful alcohol consumption-II. *Addiction*. 1993;88(6):791-804.
3. Babor TF, Higgins-Biddle JC, Saunders JB, Monteiro MG. The alcohol use disorders identification test (AUDIT): Guidelines for use in primary care. *World Heal Organ*. 2001.
4. Rutter M, Tizard J, Whitmore K. *Education, Health and Behaviour*. London: Longman.(reprinted 1981, Melbourne, FL: Krieger); 1970.
5. Rodgers B, Pickles A, Power C, Collishaw S, Maughan B. Validity of the Malaise Inventory in general population samples. *Soc Psychiatry Psychiatr Epidemiol*. 1999;34(6):333-341.

© 2026 Stott J et al. *JAMA Network Open*.

eTable 2. Missing Data Analysis

		Excluded due to missing data	Included in main model	Difference
ADHD traits, Mean (SD)		0.08 (0.93)	-0.09 (0.86)	t(14745)=10.30, *p*<.001
Multimorbidity, N (%)				
	No	1,468 (63.97)	6,807 (62.28)	$\chi^2(1)$=2.30, *p*=.13
	Yes	827 (36.03)	4,123 (37.72)	
Number of physical health problems, Mean (SD)		1.29 (1.25)	1.32 (1.24)	t(13223)=-1.21, *p*=.23
Physical health related disability, Mean (SD)		17.34 (32.88)	14.89 (30.94)	t(7925)=2.51, *p*=.01
Proportion current smoking, Mean (SD)		0.32 (0.39)	0.30 (0.38)	t(10454)=2.50, *p*=.01
Proportion high alcohol use, Mean (SD)		0.21 (0.30)	0.21 (0.29)	t(7930)=0.76, *p*=.45
Proportion high BMI, Mean (SD)		0.49 (0.41)	0.50 (0.40)	t(9209)=-1.20, *p*=.23
Proportion high distress, Mean (SD)		0.17 (0.27)	0.16 (0.26)	t(9448)=1.50, *p*=.13
Education, N (%)				
	0	485 (37.16)	1,954 (27.37)	$\chi^2(3)$=52.13, *p*<.001
	1	360 (27.59)	2,300 (32.22)	
	2	58 (4.44)	404 (5.66)	
	3	402 (30.80)	2,480 (34.74)	
Sex, N (%)				
	Male	2,351 (59.70)	5,357 (49.01)	$\chi^2(1)$=132.47, *p*<.001
	Female	1,587 (40.30)	5,573 (50.99)	
Ethnicity, N (%)				
	White	2,588 (93.09)	10,584 (96.83)	$\chi^2(1)$=82.27, *p*<.001
	Minority ethnicity	192 (6.91)	346 (3.17)	
Social class at age 10				
	Unskilled	158 (6.88)	433 (3.96)	$\chi^2(5)$=107.02, *p*<.001
	Partly skilled	376 (16.36)	1,460 (13.36)	
	Manual	1,038 (45.17)	4,476 (40.95)	
	Non-manual	205 (8.92)	1,211 (11.08)	

© 2026 Stott J et al. *JAMA Network Open*.

Managerial and technical	426 (18.54)	2,672 (24.45)
Professional	95 (4.13)	678 (6.20)

© 2026 Stott J et al. *JAMA Network Open*.

eTable 3. Health Problems Reported by Age 46 Years by Condition

Health problem by age 46		N (%)
Asthma/wheezy bronchitis		
	No	8,730 (79.87)
	Yes	2,200 (20.13)
Diabetes		
	No	10,506 (96.12)
	Yes	424 (3.88)
Epilepsy/seizure		
	No	10,682 (97.73)
	Yes	248 (2.27)
Backache/back problem		
	No	6,907 (63.19)
	Yes	4,023 (36.81)
Cancer/leukaemia		
	No	10,688 (97.79)
	Yes	242 (2.21)
Hearing problems		
	No	9,966 (91.18)
	Yes	964 (8.82)
Migraine		
	No	7,597 (69.51)
	Yes	3,333 (30.49)
Problems with stomach/bowels/gallbladder		
	No	8,404 (79.41)
	Yes	2,179 (20.59)
Problems with bladder or kidneys		
	No	9,738 (92.02)
	Yes	845 (7.98)

© 2026 Stott J et al. *JAMA Network Open*.
