# Supplementary material for: Attention-Deficit/Hyperactivity Disorder Traits in Childhood and Physical Health in Midlife
Source: JAMA Netw Open. 2026 Jan 21;9(1):e2554802. doi: 10.1001/jamanetworkopen.2025.54802 (PMC12824776; doi:10.1001/jamanetworkopen.2025.54802)
Supplement: Supplement 2. — Data Sharing Statement [file jamanetwopen-e2554802-s002.pdf]

Data Sharing Statement

Data
Data available: No

Additional Information
Explanation for why data not available: Data are already freely available via the UK Data Service
